# Cryopreservation of sessile oak (*Quercus petraea* (Matt.) Liebl.) plumules using aluminium cryo-plates: influence of cryoprotection and drying

**DOI:** 10.1186/s13007-024-01161-y

**Published:** 2024-04-12

**Authors:** Urszula Wasileńczyk, Mikołaj Krzysztof Wawrzyniak, João Paulo Rodrigues Martins, Paulina Kosek, Paweł Chmielarz

**Affiliations:** 1 Kostrzyca Forest Gene Bank, Miłków, Poland; 2 Institute of Dendrology, Polish Academy of Sciences, Kórnik, Poland

**Keywords:** Sessile oak, Plant germplasm, Desiccation, Aluminium cryoplate, Vitrification, Long-term storage, Liquid nitrogen, In vitro culture, Micropropagation, Gene banks

## Abstract

**Background Quercus:**

seeds that are recalcitrant to desiccation and freezing temperatures cannot be stored in gene banks under conventional conditions. However, the germplasm of some recalcitrant seeded species can be stored in liquid nitrogen (–196 °C). Unfortunately, for many species, among them for almost the whole genus *Quercus*, an effective cryostorage method is still unknown. In this study, we propose a successful cryostorage protocol for *Quercus petraea* (Matt.) Liebl. germplasm using plumules (a shoot apical meristem of an embryo) frozen on aluminium cryo-plates.

**Results:**

The plumules isolated from the acorns of ten provenances were prestored in 0.5 M sucrose solution (for 18 h). To form alginate beads (one plumule per bead), the plumules were placed in the wells of a cryo-plate and embedded in calcium alginate gel. For cryoprotection, the encapsulated plumules were immersed in cryoprotectant solution containing 2.0 M glycerol and different concentrations of sucrose (0.8–1.2 M) for 40 min at 25 °C and desiccated under a laminar flow cabinet for 1.0–4.0 h. Cryo-plates with plumules were directly immersed in liquid nitrogen and then cryostored for 30 min. For rewarming, cryo-plates with plumules were immersed in 1.0 M sucrose solution and rehydrated for 15 min at 25 °C. Survival rates varied from 25.8 to 83.4 were achieved after cryoprotection in 1.0 M sucrose solution and the drying of plumules for 2 h. The in vitro regrowth rate of cryopreserved plumules varied among provenances and was 26–77%.

**Conclusions:**

This study presents, for the first time, a successful, simple and effective protocol for the cryopreservation of *Q. petraea* germplasm that could be used in gene banks. The experiment was successfully repeated on seeds from various provenances, each yielding similar, good results. However, seed quality and storage time after harvesting are important factors in plumule regrowth after cryopreservation.

## Introduction

Sessile oak (*Quercus petraea* (Matt.) Liebl.) is widespread across most of Europe and is one of the most historically, economically and ecologically important broadleaved tree species [1–3]. Oak decline, which has been observed since 1982, has led to a deterioration of the health of oak stands in Europe [4], and due to extending periods of drought and/or waterlogging, their vitality continues to decline [5–7]. Moreover, the growing demand for timber further decreases species genetic diversity. Of the 500 oak species that exist worldwide, at least 20% are under conservation concern [8], especially in species diversity centres such as the Indomalayan realm, where urgent conservation efforts are needed [9]. Conservation efforts include establishing *ex situ* collections of oak genetic resources, which can provide a source of planting material for ecological restoration [10]. However, oak seeds are highly desiccation sensitive (categorized as *recalcitrant* seeds) and cannot be stored long-term at subzero temperatures [11, 12]. Attempts to desiccate oak seeds was found to cause irreversible damage, leading to a loss of viability [13]. Usually, whole acorns are short-term stored at 40% acorn moisture content at temperatures between − 1 °C and − 3 °C [14]. Under such conditions, depending on the quality of the seeds, acorns can be stored for between 6 and 24 months without losing viability [15, 16]. To ensure long-term conservation of these species *ex situ* in gene banks, special techniques such as cryopreservation must be used [12].

Cryopreservation can be an efficient method for genetic conservation based on freezing/storing plant structures such as plant cells, tissues, organs, or seeds in liquid nitrogen (LN, − 196 °C). The first step in applying the cryopreservation technique entails dehydrating water-rich cells (physical or osmotic dehydration), followed by ultrarapid freezing. Properly dehydrating plant tissues is essential for preventing intracellular water crystallization, which can lead to cell damage or even death during freezing [17].

Tissue dehydration can be performed by incubating the tissue with sugars [18]. Sucrose has been shown to be an efficient pretreatment before freezing, and its concentration may vary depending on the plant species [19]. In addition to promoting dehydration before cryopreservation, sucrose has low toxicity and is membrane impermeable [20]. It can also modulate the contents of proline and sugars present in the plant material, which may prevent protein denaturation and damage in the cell membranes [21]. Air-drying conditions may enhance the effects of sucrose dehydration on seeds, cells and embryonic axes. These conditions, can also interfere with survival after recovery from freezing [19, 22, 23].

To date, successful cryopreservation has been reported for 15 oak species, a quantity that still represents only a small percentage of the total diversity of the *Quercus* genus. Different plant materials, such as pollen, embryos (somatic and zygotic), shoot tips, the plumules, and dormant buds, have already been cryopreserved from *Quercus* spp [8]. . According to these cited authors, the embryonic axes remain the most widely used explants due to their relatively simple regrowth procedures and the high genetic variability of the material. However, the regeneration rate in complete plants after freezing is low. High regrowth from embryogenic axes has only been found in only four species of black oaks (*Q. faginea* Lam [24]. , *Q. gambelli* Nutt., *Q. rubra* L. and *Q. schottkyana* Rehder & E.H. Wilson [25]). Chmielarz et al. [10] and Plitta et al. [23] successfully employed plumules for the cryopreservation of *Q. robur*. These authors reported a high survival rate and formation of viable seedlings after freezing. In the present work, we also used plumules, which were extracted from *Q. petraea* seeds collected from different provenances.

The aim of this study was to investigate the effects of different sucrose concentrations (the best cryoprotection), air-drying time after cryoprotection and cooling (aluminium cryo-plates) on the successful cryopreservation of *Q. petraea* plumules isolated from the seeds of ten provenances located in Poland.

## Materials and methods

### Plant material

**Fig. 1:**
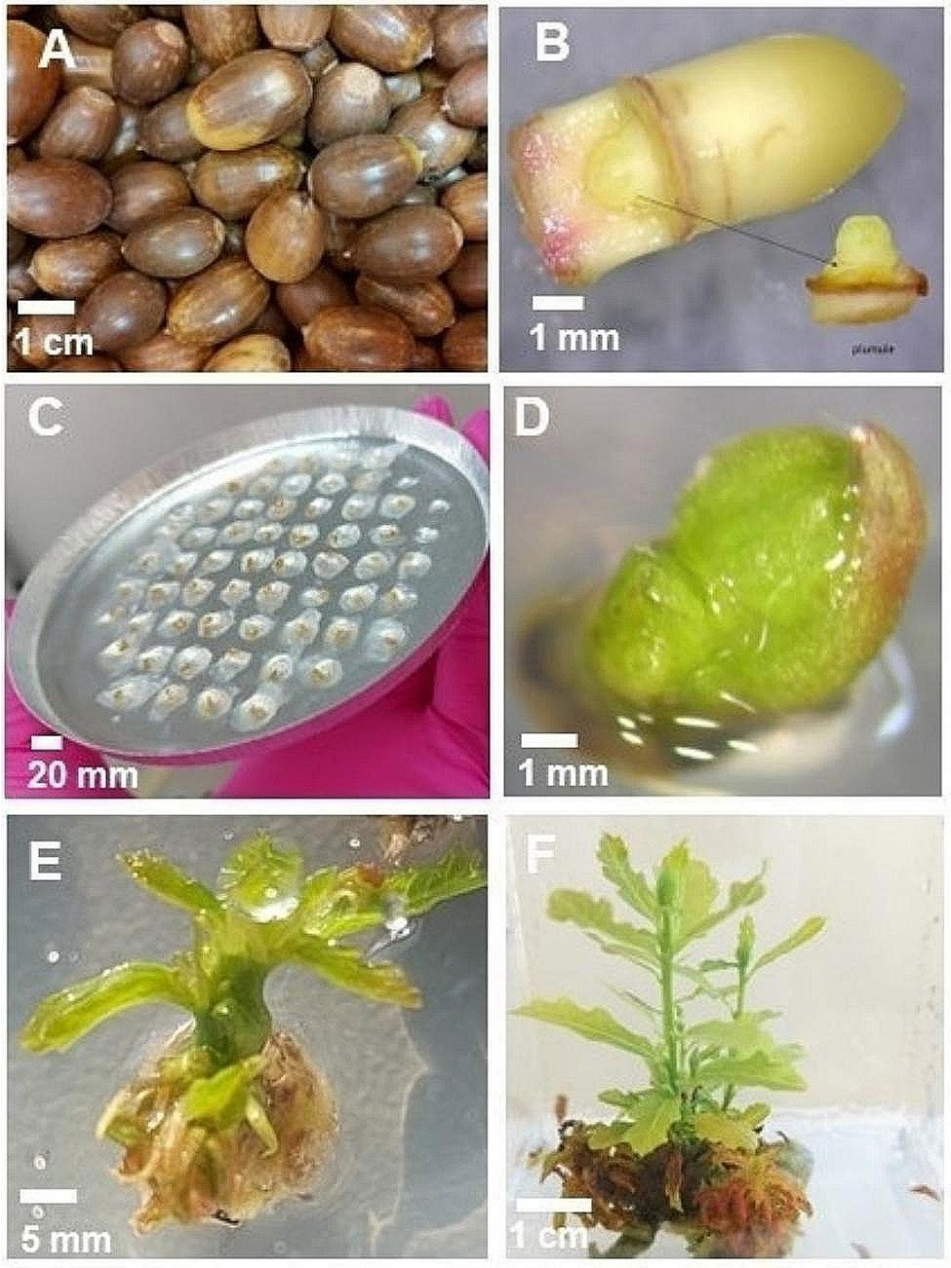
Cryopreservation of *Q. petraea* plumules and their in vitro regrowth. Acorns from which embryonic axes were isolated (A), embryonic axes from which plumules were isolated (B), an aluminium plate with plumules placed in 3 mm wells capsulated in calcium alginate (beads) - cryoprotection and desiccation stage (C), a surviving plumule after storage in liquid nitrogen, 1 week of in vitro culture (D), after 2 weeks of in vitro culture – regrowth of cryopreserved plumules (E), and 3-month-old shoots with leaves derived from plumules after cryopreservation (F)

Mature acorns of sessile oak (Fig. 1A) were collected after they had shed in October 2021 (6 provenances) and October 2022 (4 provenances) in Poland. After collection, fresh acorns with a moisture content (MC) of 40.0–45.9%, determined with a moisture analyser (Sartorius MA 45, made in Germany) (Table 1), were preliminarily stored in loosely closed, 70 L plastic barrels [26] at − 1.5 °C. The plumules (shoot meristem surrounded by leaf primordia, 1 mm in size) were excised from the acorns within 4 months after storage.

**Table 1:** Acorns were collected from 10 provenances with different levels of moisture contents (in the table, the moisture content (MC) is expressed in % according to the fresh mass of the acorn). The plumule moisture content was assessed by air-drying at 104 °C for 18 h after the plumules were stored in water for 18 h at 4 °C

Provenience			Acorns` moisture content	Plumules moisture content	Plumule weight	Number of plumules in each variant	Initial viability
			MC %	MC %	g	pcs.	%
				**2021**			
Smolarz		52º00’N 15°46’E	44.6	-	-	-	100
Jarocin		52º06’N 17°20’E	44.6	72.5	0.0025	3 × 25	100
Nieszczyce		52º32’N 16°00’E	43.7	73.1	0.0020	3 × 20	100
Łopuchówko		52º40’N 17°04’E	42.9	71.0	0.0024	3 × 25	100
Karczma Borowa		51º51’N 16°37’E	42.9	-	-	-	100
Żołędowo		53º10’N 18°01’E	40.0	-	-	-	100
				**2022**			
Syców		51°18′N 17°43′E	43.9	65.2	0.0025	20	100
Złotoryja		51°504 N 15°54′E	45.1	65.5	0.0028	30	100
Łopuchówko		52º40’N 17°04’E	44.7	62.6	0.0028	3 × 13	100
Jarocin		52º06’N 17°20’E	45.9	65.6	0.0035	3 × 20	99

#### Isolation of plumules

The surfaces of the acorns were washed in 10% commercial bleach (< 5% sodium hypochlorite NaClO and < 1% sodium hydroxide NaOH) for 15 min, rinsed with running water, sprayed with 70% alcohol and dried on a table at room temperature. Plumules were isolated under nonsterile conditions in two steps: [1] Embryonic axes were isolated from the acorns (Fig. 1B). During the isolation procedure, embryonic axes were placed on pieces of filter paper moistened with distilled water within a closed Petri dish and placed on ice. Subsequently, the plumules were isolated from the embryonic axes with a scalpel under a binocular microscope [10]. Excised plumules were preliminarily stored in a 0.5 M sucrose solution in darkness at 4 °C for 18 h [10]. The average mass of one plumule for all provenances was 0.002–0.003 g. The initial viability was examined and was found to be 100%. To determine the initial viability, three replicates of 50 plumules were used. After preliminary storage, the plumules were sterilized in 10% commercial bleach (< 5% sodium hypochlorite NaClO and < 1% sodium hydroxide NaOH) for 5 min and then rinsed three times in sterile 0.5 M sucrose solution. Plumules were cultured in sterile plastic Petri dishes (60 mm) on Murashige and Skoog (MS) medium [27]. Survival of the plumules was assessed after 2 weeks of in vitro culture. Plumules used as a control variant in this experiment were not cryoprotected and not desiccated.

#### Assessment of plumule moisture content (MC)

The plumule moisture content was assessed by air-drying at 104 °C for 18 h after the plumules were stored in water for 18 h at 4 °C.

### Cryo-plate procedure – cryoprotection, drying, and cooling

The plumules were sterilized after preliminary storage using the same procedure as for initial viability. Sterile plumules were placed on an aluminium cryo-plate 100 mm in diameter × 7 mm in depth with 50 handmade wells (3 mm in diameter, 0.8 mm in depth) for all the experimental treatments. For each treatment, 50 plumules/one cryo-plate were used in 3 replicates. The plumules were placed individually in the wells with the tip of the pipette and embedded in alginate beads by adding one drop of 2% (w/v) sodium alginate (viscosity 15–25 cp., Sigma‒Aldrich 180,947) in water (approximately 2.0 µl/well; Fig. 1C). The plumules were then immersed in sterile 0.1 M calcium chloride solution with 0.4 M sucrose on aluminium plates for 15 min to achieve complete polymerization of the alginate gel. After removing the excess calcium chloride solution with a pipette, the gel-encapsulated plumules were flooded with sterile osmoprotectant solution containing 2.0 M glycerol with different concentrations of sucrose − 0.8, 1.0 or 1.2 M [28] and soaked for 40 min at 25 °C. Subsequently, the plumules from each sucrose treatment were attached to the cryo-plate and desiccated for 1.0, 1.5, 2.0, 2.5, 3.0, 3.5 or 4.0 h at 25 °C under the air flow of a laminar flow cabinet (Aura Vertical SD 4, BioAir S.p.A).

After desiccation, the cryo-plates containing the capsulated beads were directly immersed in LN for 30 min. For rewarming, the cryo-plates were immersed in 1.0 M sucrose and incubated for 15 min at 25 °C.

### In vitro culture after cryostorage

After cryostorage, the plumules were cultured in sterile plastic Petri dishes (60 mm) on Murashige and Skoog Medium (MS) [27] (Fig. 1D) supplemented with 1.0 mg/l 6-Benzylaminopurine (BAP) [29] and sucrose (30 g/l). After one week of culture, the plumules were transferred to Woody Plant Medium (WPM) [30] supplemented with 0.8 mg/l BAP [31]. The plumules were cultured under standard conditions under light, with a 16 h/8 h light/dark photoperiod at 25 °C and a light intensity of 78 µM/m^− 2^s^− 1^. Shoots with small leaves formed bunches (Fig. 1E), so they were separated and transferred every 3–4 weeks into magenta vessels (Sigma) onto the same medium and were subcultured (Fig. 1F). The plumules were considered viable if growth was observed after 2 weeks of in vitro culture (referred to in this study as survival). Additional observations were made after 3 months of in vitro culture (referred to in this study as recovery).

### Statistical analyses

All analyses were conducted using R software (R Core Team, 2022). The survival of the plumules after cryostorage was analysed using ANOVA on a generalized linear model with a binomial function. Tukey’s test was used for *post hoc* comparisons of the means. For each treatment, 50 plumules/one cryo-plate were used in 3 replicates.

## Results

### Sucrose concentration

**Fig. 2:**
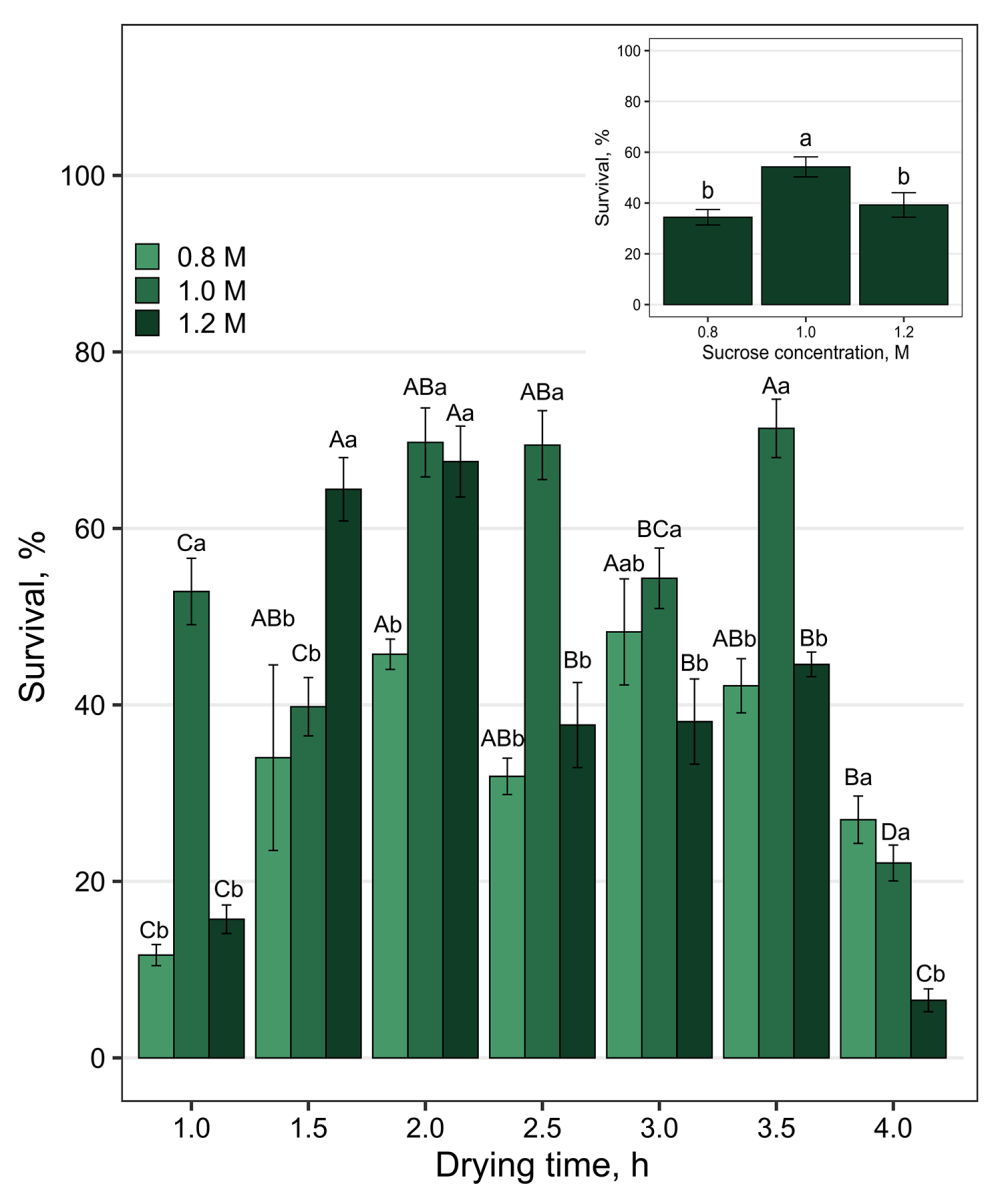
Effects of different sucrose solutions (0.8; 1.0; 1.2 M) and drying times (1.0–4.0 h) on the survival of *Q. petrea* plumules after storage in liquid nitrogen (+ LN). The initial growth of the nonstored plumules was 100% (Table 2). Bars represent the mean ± SE; bars marked with different letters differ significantly (Tukey’s tests *p* ≤ 0.05); capital letters indicate differences between the same concentrations at different times and small letters indicate differences between different concentrations at the same time

Both the time of desiccation and the sucrose concentration had a significant effect on the survival of the cryopreserved plumules. The highest survival after cryopreservation was obtained in the plumules treated with 1.0 M (54%; Fig. 2) sucrose solution. Cryoprotection of plumules with a 0.8 or 1.2 sucrose concentration resulted in plumule survival after thawing 34–39%.

### Drying time

The highest plumule survival was observed after 2, 2.5 and 3.5 h of drying (69–71%; Fig. 2), with a sucrose concentration of 1.0 M. The plumules dried for 4 h had significantly lower regrowth, at 22%. Additionally, short drying resulted in lower plumule survival, at a rate of 52% after 1 h and 39% after 1.5 h.

Plumules cryoprotected in 1.2 M sucrose dried more rapidly than those cryoprotected in lower concentrations of sucrose. The highest survival was observed after 1.5 and 2 h of drying, with 64 and 67% survival, respectively, in comparison with that after 1 or 4 h of drying (16 and 7%, respectively) (Fig. 2).

In general, the most effective regrowth of cryopreserved plumules was found after 2 h at each tested concentration of 0.8, 1.0 and 1.2 M sucrose, yielding 45.74, 69.75 and 67.57%, respectively. (Fig. 2). For the 1.0 M sucrose concentration, better survival results were obtained after a 0.5 h longer drying time than after a 1.2 M sucrose concentration, which was 70% for 2.0 h, 69% for 2.5 h, and for 1.2 M sucrose, which were 64% for 1.5 h and 68% for 2.0 h, respectively, and which was also associated with the need for longer drying of plumules treated with a sucrose solution at a lower concentration.

### Regrowth

Approximately 100% of the plumules survived after isolation and before freezing, which was determined on the day of the experiment. (Table 1). However, the survival of the examined provenances was strongly dependent on seed quality, which decreased after 3 months of storage (Table 2) and was not correlated with plumule weight (Cor = − 0.35, *p =* 0.44). Chmielarz et al. [10] drew attention to the relationship between plumules survival after cryopreservation and the time of acorns storage after harvest. This is probably due to several stress factors such as osmotic shock, lipid peroxidation or damage to cell membranes that occur during seed storage [32]. The highest survival was found for Syców 2022, Jarocin, Łopuchówko and Złotoryja 2022, with 65–83% (Table 2). Survival in Jarocin 2022, Karczma Borowa, Nieszczyce and Łopuchówko 2022 ranged from 52 to 62%. The lowest plumule survival was recorded for the Smolarz and Żołędowo provenances, at 26% and 37%, respectively (Table 2). The recovery rates of cryopreserved plumules compared with the survival rates decreased or remained at the same level. The largest decrease, from 75% survival to 58% recovery, was observed for the seeds of the Złotoryja provenance.

**Table 2:** Effect of *Q. petraea* provenances on plumule survival and recovery after cryoprotection (1.0 M sucrose + 2.0 M glycerol) followed by storage in liquid nitrogen (+ LN). Mean ± SD; The highest survival rate of the plumules was obtained from acorns that were stored for up to 3 months

Provenience	Survival (%)	Recovery (%)
Jarocin (2022)	52.1 ± 10.1	50.9 ± 8.0
Jarocin	65.3 ± 8.1	58.2 ± 7.6
Łopuchówko (2022)	62.3 ± 11.7	62.3 ± 11.7
Łopuchówko	83.4 ± 5.6	77.2 ± 5.8
Syców (2022)	68.8 ± 13.0	59.1 ± 3.7
Złotoryja (2022)	75.1 ± 2.5	58.2 ± 8.9
Nieszyce*	60.4 ± 7.2	60.4 ± 7.2
Smolarz*	25.8 ± 1.3	25.8 ± 1.3
Żołędowo*	36.8 ± 6.5	35.5 ± 4.4
Karczma Borowa*	54.1 ± 11.5	50.9 ± 12.0

* - acorns stored for more than 3 months in loosely closed, 70 L plastic barrels [26] at − 1.5 °C.

## Discussion

Cryopreservation of embryogenic axes or plumules isolated from recalcitrant seeds has proven to be successful in many species, such as the Brazilian green dwarf coconut (*Cocos nucifera* L.) plumules [33], chestnut [34] and several other citrus species [35]. Recently, Ballesteros and Pence [36] reported the regeneration of *Aesculus hippocastanum, A. glabra* and *Juglans nigra* embryo axes after 23 years of storage in LN. In 2011, Chmielarz and coworkers described the possibility of cryogenic storage of pedunculate oak plumules, obtaining a survival rate of 60% and regrowth of ca. 25%. Relatively slow in vitro regrowth has greatly limited the use of the described methodology on a wider scale in gene banks [37]. Our results presented here show that *Q. petraea* plumules can be successfully stored in liquid nitrogen, with a high survival rate of 83% and recovery of 77%, depending on seed quality, using modified methods with aluminium cryo-plates. Our method significantly improved survival in comparison with the other methods. For example, shoot tips of 4 oak species were cryopreserved using droplet vitrification and recovered on WPM supplemented with 0.2 mg/L BAP, 3% sucrose, and 0.25% Gelzan. Survival after LN exposure differed by species, ranging from good survival with *Q. virginiana* (55%) to poor survival with *Q. hinckleyi* (18%) and from *Q. suber* (15%) to no survival with *Q. gambelii* [38]. In general, cryopreservation of the embryonic axes of *Quercus* spp. is the preferred method for *ex situ* conservation. However, the response to water loss among the embryonic axes of oaks is variable, impacting recovery after LN exposure. Ballesterose and coworkers [39] investigated embryonic axes in vitro survival in 12 *Quercus* species from the UK, Spain and Lebanon. Embryonic axes with a small portion (1 mm) of cotyledon attached were partially (“flash”) dried and exposed to LN after treatment with ascorbic acid after excision. These authors observed that the differential pattern of water loss affected recovery after exposure to LN, resulting in most of the species having better root survival. Fast drying and cooling were relatively successful for the cryopreservation of *Q. pyrenaica*, *Q. rubra, Q. ilex* and *Q. coccifera* embryos (root + shoot recovery between 25 and 50%), but not for the other species (root + shoot recovery). The lower survival rate of the plumules treated with a solution containing 0.8 M sucrose concentration is related to the higher water content of the plumules, which significantly affects the survival rate of the plumules after freezing. After treatment with a concentration of 0.8 M sucrose, plumule regrowth was highest after 3 h of drying, reaching 48%. Similar results were obtained after 1.5, 2 and 3.5 h of drying, and significant differences were detected only at 1.0 and 4.0 h. Sucrose is typically used as an osmoprotectant, which enhances plant tolerance to dehydration stress by maintaining turgor pressure [40]. Plumules cryoprotection in the cryoprotectant solution is the first stage of their desiccation. Thus, plumules treated with a higher concentration of sucrose (more desiccated) require a shorter drying time compared to plumules treated with a lower concentration of sucrose solution. Moreover, in contrast to other cryoprotectants, such as dimethyl sulfoxide (DMSO), sucrose is membrane impermeable and has low toxicity [20]. However, the osmotic dehydration step may occur rapidly at high concentrations of sucrose or gradually over time at lower concentrations. The correct procedure depends heavily on the tissue in hand [41]. In some species of chrysanthemum, the dehydration time might vary between 3 and 9 h [42, 43]. The success of postcryogenic tissue survival depends on the selection and optimization of the correct cryopreservation procedure, minimizing exposure to stress (i.e., oxidative stress) and cellular damage [44]. However, the application of cryopreservation methods is still limited, as they need to be refined for individual species. In our research, the experiments conducted aimed to test whether the freezing of explants on aluminium plates [45, 46] would also work for cryopreservation of *Q. petraea* plumules. Cryopreservation of plumules is essential due to the small size of the plumules and the presence of many meristematic cells [47]. The advantage of this method is the possibility of rapid cooling and heating to avoid lethal ice formation in cells, which can be achieved by using aluminium cryo-plates. In addition, aluminium plates have a very high thermal conductivity, which allows rapid cooling [28, 48] and protects the plumules from damage during their transfer through the different steps of the cryoprotection procedure, eliminating physical damage to the tissue by not touching them until the explants are placed on the medium. A similar method was used by Yamamoto et al. [49] and Tanaka et al. [46] for cryopreservation of *Solanum tuberosum* L. shoot tips. They described two successful (almost 100% regrowth) rapid freezing steps of shoot tips on aluminium cryo-plates after dehydration in vitrification solution followed by dehydration under laminar flow. A similar method using aluminium cryo-plates involving encapsulation and osmoprotection (2.0 M glycerol and 0.6 M sucrose), and air desiccation was used by Tanaka et al. [46] for mulberry (*Morus alba*) cryopreservation with almost 90% survival after thawing. The effect of the size of shoot tips on regrowth after cryopreservation was also crucial [49]. According to these authors, the smaller the size of shoot tips, the higher the survival rate is [49]. This was in accordance with the study of mulberry shoot tips [46], whose size before freezing did not exceed 1.5 mm. In our research, the size of the *Q. petraea* plumules was also relatively small and did not exceed 0.2 mm on average for plumules derived from seeds of the 10 provenances. Their weight was not correlated with survival.

## Conclusion

*Q. petraea* plumules cryopreserved using a modified aluminium cryo-plate method developed in this study survived at a high rate (up to 83%). The experiment was successfully repeated on seeds various provenances, each yielding similar, good results. However, seed quality and storage time after harvesting are important factors in plumule regrowth after cryopreservation. Properly growing seedlings with a shoot and a root were derived from plumules after cryopreservation.

Based on the results obtained, the optimal cryo-plate procedure for *Q. petraea* plumules is as follows:

**Fig. 3:**
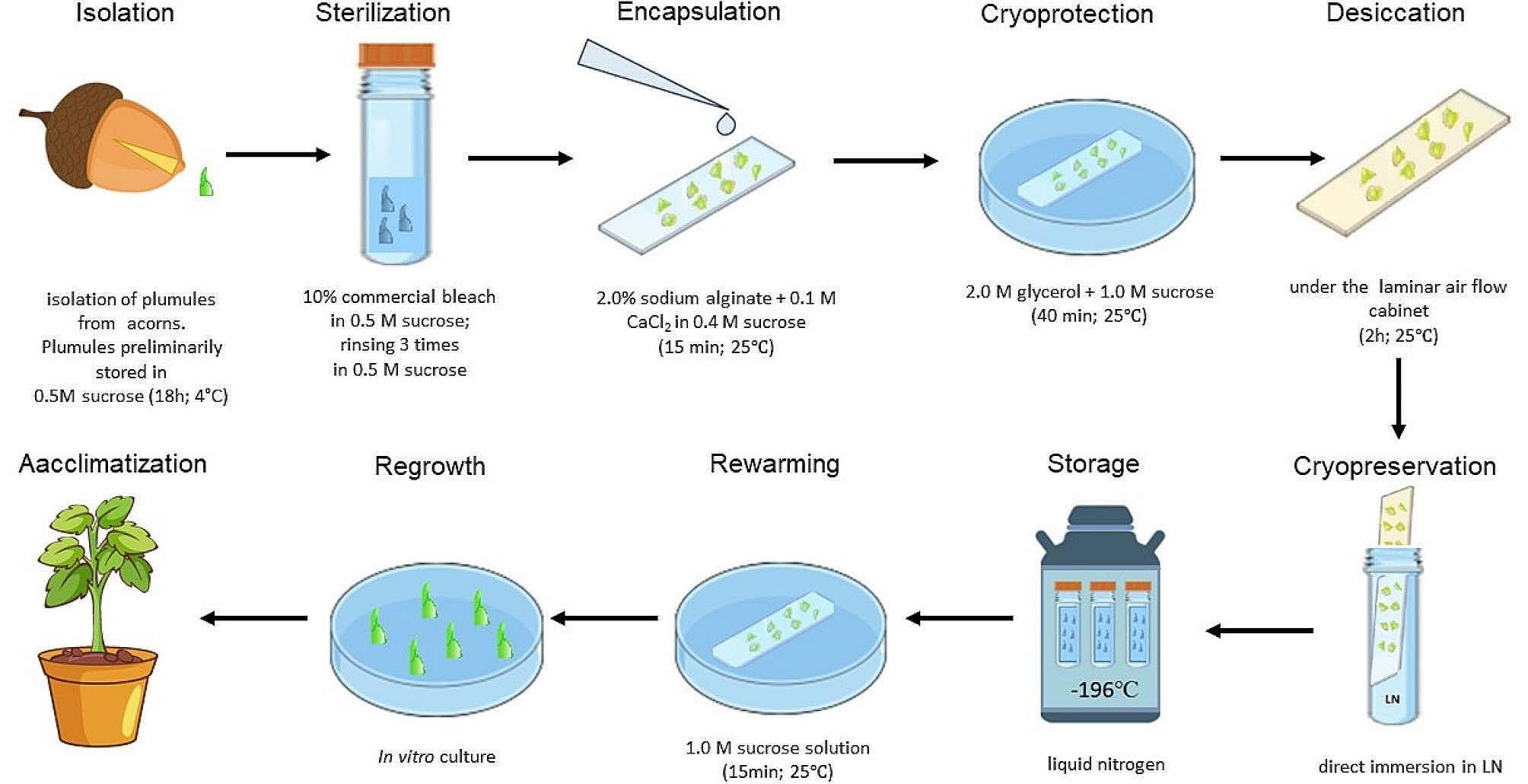
The optimal aluminium cryo-plate procedure for *Q. petraea* plumules cryopreservation

Isolated plumules (1.0 – 1.5 mm) were attached to the cryo-plate by calcium alginate gel. Then, osmoprotection of the plumules is performed by immersing the plumules together with the cryo-plate in solution containing 2.0 M glycerol and 1.0 M sucrose for 40 min at 25 °C. Next, the plumules on the cryo-plate were dehydrated under a laminar air flow cabinet for 2.0 h at 25 °C. The cryo-plate with plumules is plunged directly into the LN (Fig. 3.). Regrowth was tested for materials from10 provenances, and seed lots collected from 8 out of 10 locations demonstrated more than 50% survival and regrowth after cryopreservation using this method, ranging from 26 to 77%.

## Data Availability

The datasets generated and analysed during the current study are available in the RepOD repository. https://doi.org/10.18150/LLHKEX.
